# The adsorption features between insecticidal crystal protein and nano-Mg(OH)_2_

**DOI:** 10.1098/rsos.170883

**Published:** 2017-12-06

**Authors:** Xiaohong Pan, Zhangyan Xu, Yilin Zheng, Tengzhou Huang, Lan Li, Zhi Chen, Wenhua Rao, Saili Chen, Xianxian Hong, Xiong Guan

**Affiliations:** 1 State Key Laboratory of Ecological Pest Control for Fujian and Taiwan Crops and Key Lab of Biopesticide and Chemical Biology, Ministry of Education, College of Plant Protection, College of Resources and Environmental Sciences, Fujian Agriculture and Forestry University, Fuzhou, Fujian 350002, People's Republic of China; 2 Fujian-Taiwan Joint Center for Ecological Control of Crop Pests, Fuzhou, Fujian 350002, People's Republic of China

**Keywords:** insecticidal protein, nano-Mg(OH)_2_, adsorption isotherm, adsorption kinetics, adsorption thermodynamics

## Abstract

Nano-Mg(OH)_2_, with low biological toxicity, is an ideal nano-carrier for insecticidal protein to improve the bioactivity. In this work, the adsorption features of insecticidal protein by nano-Mg(OH)_2_ have been studied. The adsorption capacity could reach as high as 136 mg g^−1^, and the adsorption isotherm had been fitted with Langmuir and Freundlich models. Moreover, the adsorption kinetics followed a pseudo-first or -second order rate model, and the adsorption was spontaneous and an exothermic process. However, high temperatures are not suitable for adsorption, which implies that the temperature would be a critical factor during the adsorption process. In addition, FT-IR confirmed that the protein was adsorbed on the nano-Mg(OH)_2_, zeta potential analysis suggested that insecticidal protein was loaded onto the nano-Mg(OH)_2_ not by electrostatic adsorption but maybe by intermolecular forces, and circular dichroism spectroscopy of Cry11Aa protein before and after loading with nano-Mg(OH)_2_ was changed. The study applied the adsorption information between Cry11Aa and nano-Mg(OH)_2_, which would be useful in the practical application of nano-Mg(OH)_2_ as a nano-carrier.

## 1. Introduction

*Bacillus thuringiensis* (Bt), a spore-forming Gram-positive strain, is widely used as a biopesticide, and can form insecticidal crystal protein (including Cry and Cyt proteins) during the stationary phase of growth [1–4]. But there are some disadvantages when the Bt product is applied in the field, such as the active ingredients being rapidly inactivated by ultraviolet (UV) light or degraded by other microorganisms [5–7], which hinder its effective application. There are several ways to prevent its inactivation and degradation, such as encapsulation, adding a coating agent and ultraviolet stabilizers, or gene regulation [8–11]; however, the additives have poor compatibility or stability, which might increase risk to the environment of the biopesticide.

In recent years, numerous studies have focused on nanomaterials because nanoparticles possess strong adsorption capacity, high specific surface area and specific physicochemical properties [12–14]. Nanoparticles can be used as excellent nano-carriers in nanodrug delivery systems, which can improve the utilization rates and promote direct drug diffusion [12,15]. Additionally, nanoparticles are also widely used for biopesticides. It was reported that porous hollow silica nanoparticle (PHSN) carriers exhibited remarkable UV-shielding properties for avermectin, and the avermectin could be released slowly after loading on PHSN carriers [16]. Qin *et al*. [12] prepared nanoscale chitinases by immobilizing Bt chitinases onto the surface of silica nanoparticles through electrostatic adsorption and covalent binding; the nanoparticles could synergistically enhance the nematicidal effect, which implied that silica nanoparticles can serve as an efficient nano-carrier. Also, mesoporous silicon particles have been applied in the protection and delivery of the anthelmintic protein Cry5B [17]. Most of the previous reports aim to improve the properties of the biopesticide by effects such as UV shielding and control of release and biological activity. However, it is also essential to understand the adsorption features, because the adsorption behaviour will reflect the adsorption affinity and efficiency, and regulation of the adsorption process might enhance the insecticidal activity and facilitate the practical application of nanomaterials in biopesticide use.

Nano-Mg(OH)_2_ is an environmentally friendly material; it is widely used as an adsorbent in wastewater removal [18–20] with low biological toxicity [21,22]. Our previous studies indicated that Cry11Aa protein had high activity against disease vector mosquitoes, and the Cry11Aa protein loaded onto nano-Mg(OH)_2_ had even stronger toxicity toward mosquitoes [23]. But the adsorption features of Cry11Aa by nano-Mg(OH)_2_ still need further study in order to develop a new mosquitocide. Herein, we applied adsorption experiments (such as adsorption isotherms, kinetic and thermodynamics analysis) to investigate the adsorption characteristics of nano-Mg(OH)_2_ toward insecticidal protein. The understanding of the adsorption behaviour might be useful to regulate the adsorption in practical applications.

## 2. Material and methods

### 2.1. Cry11Aa and nano-Mg(OH)_2_

The nano-Mg(OH)_2_ was synthesized by coprecipitation of magnesium chloride hexahydrate (MgCl_2_·6H_2_O) and sodium hydroxide (NaOH) as described in previous studies [24] with minor modification. Briefly, 0.70 g MgCl_2_·6H_2_O was added into 10 ml ddH_2_O for dissolution, and 0.28 g NaOH was dissolved in 10 ml ddH_2_O, then the dissolved NaOH was slowly added into MgCl_2_·6H_2_O solution with continuous stirring overnight. Finally, the mixture was centrifuged at 10 000*g* for 5 min and washed with ddH_2_O three times for further characterization. The Cry11Aa was extracted from Bt LLP29 strain according to the method described by Helassa [25].

### 2.2. Adsorption isotherms

The adsorption isotherm experiments were performed at 4°C. The concentration of Cry11Aa ranged from 0.068 to 0.68 mg ml^−1^, and the concentration of nano-Mg(OH)_2_ was set at 10 g l^−1^. Experiments were performed with ultrasonication for 30 min, which was found to be sufficient time for the mixture to reach adsorption equilibrium. After equilibrium, the supernatant was extracted by centrifugation. Then the concentration of Cry 11Aa protein was measured, and the adsorption capacity was calculated.

### 2.3. Adsorption kinetic analysis

Nano-Mg(OH)_2_ 0.01 g was added to 1 ml protein solution (0.68 mg ml^−1^) with ultrasonication at 4°C. For time series experiments, 50 µl aliquots were extracted at the appropriate time intervals, centrifuged at 6000*g* for 3 min and the supernatant was recovered. The remaining protein concentration was measured.

### 2.4. Adsorption thermodynamics

Nano-Mg(OH)_2_ 0.01 g was added to 1 ml of 0.68 mg ml^−1^ protein solution and ultrasonicated for 2 h at a series of fixed temperatures (20°C, 25°C and 30°C) until adsorption equilibrium was reached. The protein concentration of the supernatant was measured and the adsorption capacity of was calculated.

### 2.5. The desorption experiment

The desorption experiment was conducted in a simulated natural environment (pH = 5.6). 1 ml of 30 mM MES (C_6_H_13_NO_4_S) medium was added to the loaded sample (Cry11Aa-Mg(OH)_2_) in a 2 ml centrifuge tube, and the sample was incubated at room temperature with gentle agitation. Then the supernatant was collected every 12 h by centrifugation followed by addition of fresh MES medium, for a total of seven times. The concentration of Cry11Aa protein in the supernatant was measured in order to calculate the desorption rate of Cry11Aa from nano-Mg(OH)_2_.

### 2.6. FT-IR, zeta-potential and circular dichroism analyses

The nano-Mg(OH)_2_ after Cry protein loading (0.01 g nano-Mg(OH)_2_ : 1 ml protein) was separated into supernatant and precipitate by centrifugation, and the precipitate was dried by freeze drying at −80°C. Then the nano-Mg(OH)_2_ and nano-Mg(OH)_2_ loaded with protein were prepared in a KBr pellet with a sample/KBr ratio of 1:100, and the thin pellets were analyzed using a PerkinElmer Spectrum One FT-IR spectrometer in the range of 4000–400 cm^−1^.

The surface charge of the nanoparticles before and after protein loading was measured in 50 mM citrate buffer with a Malvern Instruments Zetasizer ZS90 instrument. For each sample, an appropriate amount of undiluted solution was placed into the cuvette, and an average zeta potential value was obtained from three individual measurements. The solution media was set as water for all zeta potential measurements.

The Brunauer-Emmett-Teller (BET) surface area of the nano-Mg(OH)_2_ was measured using a Micrometrics ASAP 2020 system. The BET surface area, using the adsorption data, was determined by a multipoint BET method, and the relative pressure (P/P^0^) ranged from 0.01 to 1.0.

The circular dichroism (CD) spectra of Cry11Aa protein before and after loading onto nano-Mg(OH)_2_ were recorded on a CD spectropolarimeter (Chirascan^TM^, Applied Photophysics Ltd, UK). The samples were measured in a 1 mm pathlength quartz cell at 37°C. The spectra were scanned between 195 and 260 nm, three scans were accumulated at a scanning speed of 60 nm min^−1^ and a time constant of 0.5 s, and the spectra were averaged over three consecutive scans.

## 3. Results

### 3.1. Effect of protein concentration and adsorption isotherm study

**Figure 1.**
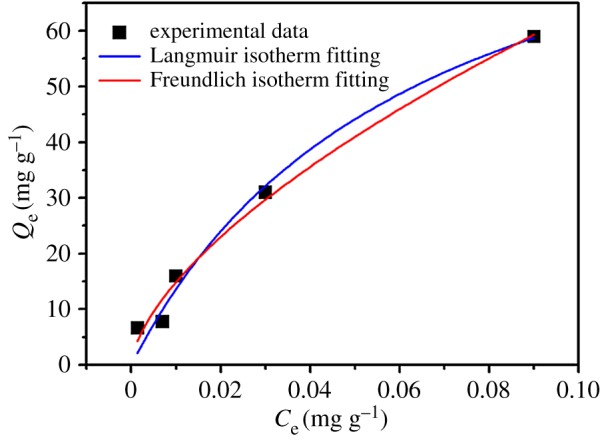
Adsorption isotherm experiment and the fitting result by Langmuir and Freundlich isotherm models for Cry protein adsorption by nano-Mg(OH)_2_.

The adsorption capacity between Cry11Aa and nano-Mg(OH)_2_ was evaluated by equilibrium experiments (figure 1), and the Langmuir and Freundlich models were applied to understand the adsorption mechanism. The calculations were described as follows:

3.1 $$\begin{array}{r} \text{Langmuir}:\;Q_{e}=\frac{Q^{0}bC_{e}}{1+bC_{e}} \end{array}$$

3.2 $$\begin{array}{r} \text{Freundlich}:\;Q_{e}=k{C_{e}}^{\frac{1}{n}} \end{array}$$

where *Q*_e_ represents the adsorbed capacity at equilibrium, *Q_0_* is maximum amount or the saturated adsorption amount (mg g^−1^), *C*_e_ is the equilibrium concentration in the solution (mg l^−1^), *b* is the Langmuir constant (l/mg) which relates to the binding strength, and *n* and *k* are the Freundlich constants which relate to the biosorption intensity and biosorption capacity, respectively.

As revealed in table 1, the Langmuir and Freundlich isotherm models are both appropriate to depict the adsorption process based on *R^2^* value, and the saturated adsorption capacity of Cry protein was 100.2 mg g^−1^ (*Q^0^*). Meanwhile, the relative larger value of the *b* constant indicated a favourable adsorptive process of Cry protein on nano-Mg(OH)_2_ according to the Langmuir fitting results, which was also confirmed by the Freundlich constant *n* (*n* lies between 1 and 10, indicating that the adsorbate is favourably adsorbed [26]).

**Table 1.** The Langmuir and Freundlich isotherm model constants for adsorption of Cry11Aa by nano-Mg(OH)_2._

	*Q^0^* (mg g^−1^)		*b* (l mg^−1^)		
Langmuir	value	s.e.	value	s.e.	*R*^2^
	100.20	16.95	15.74	5.12	0.98

	*k*		*n*		
Freundlich	value	s.e.	value	s.e.	*R*^2^
	270.54	44.40	1.59	0.14	0.98

### 3.2. Effect of time and adsorption kinetics study

**Figure 2.**
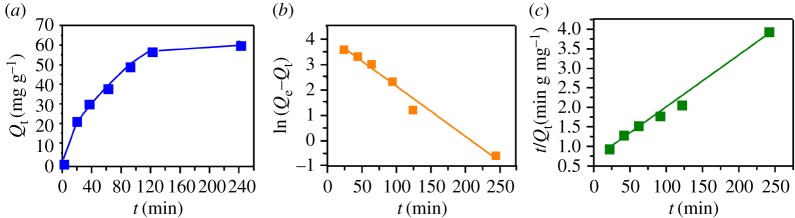
(*a*) Adsorption of Cry11Aa by nano-Mg(OH)_2_ as a function of contact time (Cry concentration 0.68 g/l, nano-Mg(OH)_2_ dosage 10 g/l); (*b*) pseudo-first order plot; (*c*) pseudo-second order plot.

The adsorption capacity between Cry11Aa and nano-Mg(OH)_2_ was evaluated by adsorption kinetics experiments. Figure 2*a* indicated that the rate of Cry11Aa adsorption by nano-Mg(OH)_2_ is rapid in the first 2 h. The adsorption of protein followed a gradual increase approaching the adsorption equilibrium, and the adsorption capacity could reach as high as 60.6 mg g^−1^. Meanwhile, pseudo-first (figure 2*b*) and -second order models (figure 2*c*) were used to evaluate the experimental data, and the kinetic parameters calculated by linear regression are given in table 2. The regression coefficients (*R^2^*) for the pseudo-first and -second order models are around 0.98, indicating that both the pseudo-first and -second order equations can fit well with the experimental data. In addition, the examined adsorption capacity (60.6 mg g^−1^) was closer to the *Q*_e_ values which were calculated by the pseudo-first and -second order kinetic models (62.30 and 75.36 mg g^−1^).

**Table 2.** The pseudo-first order kinetic and pseudo-second order kinetic constants for adsorption of Cry11Aa by nano-Mg(OH)_2._

	*Q*_e_ (mg g^−1^)	*k* g/(mg·min)	*R^2^*
first-order kinetic	62.30	0.0198	0.975
second-order kinetic	75.36	2.56 × 10^−4^	0.986

The pseudo-first (3.3) and -second (3.4) order kinetic models are expressed as:

3.3 $$\begin{array}{r} \text{ln}(Q_{e}-Q_{t})=\text{ln}\;Q_{e}-k_{1}\;t \end{array}$$

3.4 $$\begin{array}{r} t/Q_{t}=1/k_{2}{Q_{e}}^{2}+t/Q_{e} \end{array}$$

where *Q_e_* and *Q_t_* are the adsorbed amount of protein at equilibrium or at any time (mg g^−1^), respectively; *k_1_* (min^−1^) and *k_2_* (g mg^−1^ min^−1^) represent the pseudo-first and -second order rate constants.

### 3.3. Effect of temperature and adsorption thermodynamic study

**Figure 3.**
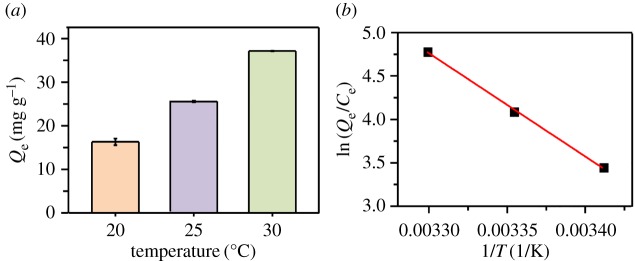
**(***a*) Effect of temperature on Cry protein loading, ±s.d. shown by the error bar. (*b*) Plots of ln (*Q*_e_/*C*_e_) versus 1/*T* for the adsorption of Cry11Aa onto nano-Mg(OH)_2_.

Additionally, the influence of different temperatures on the adsorption process was carried out. As shown in figure 3*a*, the adsorption capacity (*Q*_e_) gradually increased from 16.31 mg g^−1^ to 37.08 mg g^−1^ with an increase in temperature from 20°C to 30°C. Meanwhile, enthalpy (Δ*H*, kJ/mol) and entropy (Δ*S*, J/mol·K) were obtained from the slope and intercept of the plot from the Van't Hoff equation (as depicted in figure 3*b*), and Gibbs free energy (Δ*G*, kJ/mol) of the present batch adsorption process was calculated from the Gibbs–Helmholtz equation [26,27]:

3.5 $$\begin{array}{r} \text{ln}\;\left(\frac{Q_{e}}{C_{e}}\right)=\frac{\Delta S}{R}-\frac{\Delta H}{RT} \end{array}$$

3.6 $$\begin{array}{r} \Delta G=\Delta H-T\Delta S \end{array}$$

where *Q*_e_ is the adsorbed capacity at equilibrium (mg g^−1^), *C*_e_ is the equilibrium concentration of Cry protein in the solution (mg l^−1^), *Q*_e_/*C*_e_ is the equilibrium constant (l/g), *R* is the universal gas constant (8.314** **J mol^−1^· K) and *T* is the absolute temperature (K).

As shown in table 3, the values of Δ*G* at different temperatures are negative values, which implies that the adsorption process is spontaneous and favourable at 20°C to 30°C [27]. Meanwhile, the negative value of Δ*H* indicates the exothermic nature of the adsorption, and the Cry protein adsorption is more efficient at 30°C due to the decrease of Δ*G* that occurs with the rise of temperature [26]. Moreover, we also conducted the adsorption between Cry protein and Mg(OH)_2_ at high temperature (50 and 60°C), and the result indicated that the adsorption capacity was gradually decreased (from 23.95 mg g^−1^ to 3.13 mg g^−1^). This implies that the high temperatures are not suitable to adsorption, because the protein may be partially degraded at high temperatures. Therefore, the adsorption temperature should be controlled below 60°C according to the adsorption thermodynamic study.

**Table 3:** Thermodynamic parameters for the adsorption of Cry11Aa on nano-Mg(OH)_2_ at different temperatures.

		Δ*G* (kJ/mol)		
Δ*H* (kJ/mol)	Δ*S* (J/mol· K)	293 K	298 K	303 K
−98.63	365.07	−205.60	−207.42	−209.25

### 3.4. The desorption of Cry11Aa protein from nano-Mg(OH)_2_

**Figure 4.**
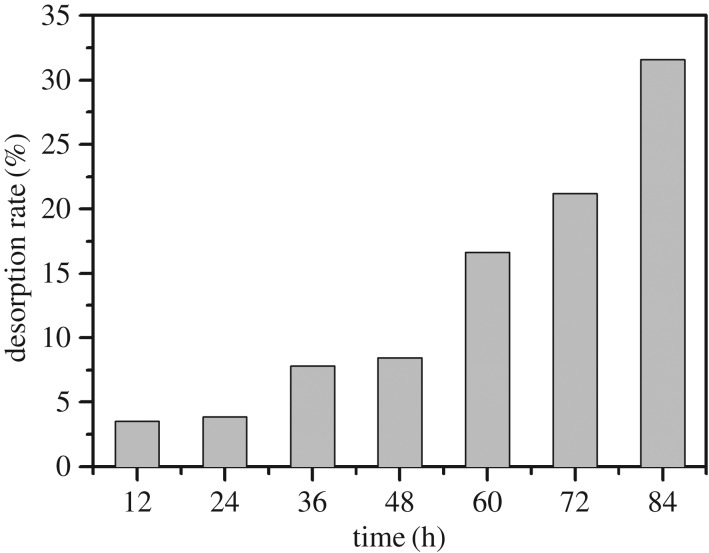
The desorption experiment of Cry11Aa protein from nano-Mg(OH)_2_ in MES medium. The MES medium was replaced every 12 h, seven times.

In order to evaluate the stability of adsorption of Cry11Aa by nano-Mg(OH)_2_, a desorption experiment was conducted. Because the loaded complex is intended for practical application in the field, MES was selected as the desorption medium in order to simulate the pH value in the natural environment (pH = 5.6). Figure 4 indicates that the Cry11Aa had stably adsorbed onto the nano-Mg(OH)_2_ during the seven cycles, and the desorption rate gradually increased from 3.5% to 31.6%. Afterwards the adsorption capacity gradually reduced, which might be due to a decrease of nano-Mg(OH)_2_ quantity in the acidic MES medium.

### 3.5. The functional group, protein structure and zeta potential analysis

**Figure 5.**
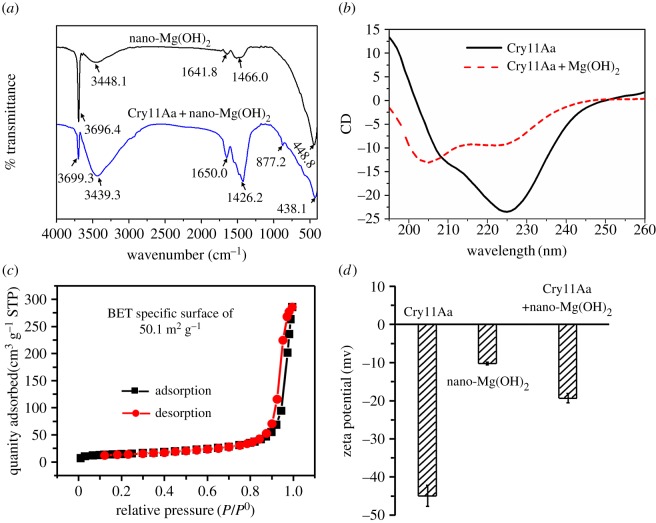
(*a*) FT-IR spectroscopic analysis of nano-Mg(OH)_2_ and Cry11Aa-Mg(OH)_2_. (*b*) CD spectra of Cry11Aa proteins before and after loading with nano-Mg(OH)_2_. The CD spectra of Cry11Aa were obtained after the subtraction of water CD, while the CD spectra of Cry11Aa loading with nano-Mg(OH)_2_ were obtained after the subtraction of nano-Mg(OH)_2_ CD. (*c*) N_2_ adsorption-desorption isotherms of nano-Mg(OH)_2_ at 77.5 K and (*d*) zeta-potential of Cry11Aa, nano-Mg(OH)_2_ and Cry11Aa-Mg(OH)_2_.

To elucidate the possible interface bonding between nano-Mg(OH)_2_ and Cry11Aa protein, FT-IR spectra were recorded from 4000 cm^−1^ to 400 cm^−1^ (figure 5*a*). For nano-Mg(OH)_2_, the absorption peaks at 3696.4 cm^−1^ and 448.8 cm^−1^ are the characteristic peaks of Mg–O [28,29], and the absorption peak at 3448.1 cm^−1^ was characterized as the stretching vibration peaks of hydroxyl groups (–OH) from water molecules [28]. Additionally, a weak peak at 1641.8 cm^−1^ was attributed to the bending vibration of water molecules adsorbed on the surface of nano-Mg(OH)_2_ [28,30], and the absorption peak at 1460–1400 cm^−1^ was also assigned to the Mg–O stretching vibrations or the Mg–O–Mg deformation vibrations [30]. Compared to unloaded nano-Mg(OH)_2_, in addition to the characteristic stretching vibration peaks of –OH (3439.3 cm^−1^) and Mg–O groups (3699 and 438 cm^−1^) of nano-Mg(OH)_2_, there was a peak at about 1650 cm^−1^ that may be assigned to the –NH_2_ groups in the loaded sample, which indicates that the Cry protein was adsorbed on the nano-Mg(OH)_2_. Meanwhile, the intensity of the Mg–O absorption peak (3699 cm^−1^) decreased significantly, and the weak peak at 877.2 cm^−1^ might belong to the aromatic C–H. Overall, all these findings confirmed that the protein was adsorbed on the nano-Mg(OH)_2_.

The secondary structure composition of the Cry11Aa protein before and after loading with nano-Mg(OH)_2_ was further analyzed using circular dichroism (CD) spectroscopy. As shown in figure 5*b*, the Cry11Aa had negative bands at 225 and 209 nm, which is close to the representative pattern of α-helical-rich proteins [31]. But after loading with nano-Mg(OH)_2_, the representative pattern of Cry protein was shifted to lower wavelength, which implies the secondary structure of protein was changed compared with the untreated Cry11Aa protein.

Figure 5*c* shows the N_2_ adsorption--desorption isotherm results at 77.5 K. The type IV isotherm curve with a clear type H_2_ hysteresis loop suggests the presence of mesopores [32], and the specific surface area of nano-Mg(OH)_2_ was 50.1 m^2^ g^−1^ as calculated using a multipoint BET.

The zeta-potential was employed to confirm the interface bonding between nano-Mg(OH)_2_ and Cry protein. As shown in figure 5*d*, nano-Mg(OH)_2_ carried negative charges and after incubation of nano-Mg(OH)_2_ with the highly negatively charged Cry11Aa protein the mixture solution still maintained a negatively charged zeta potential. This result indicates that the binding between nano-Mg(OH)_2_ and protein could not be driven by electrostatic interactions but might be by covalent linkage, and the decreased potential of nano-Mg(OH)_2_ loaded with protein also confirmed the protein was adsorbed on the nano-Mg(OH)_2_.

## 4. Discussion

The above results have shown that nano-Mg(OH)_2_ has excellent adsorption ability for Cry11Aa protein. Specifically, the equilibrium concentration was lower than 0.09 mg ml^−1^ as the initial protein concentration was 0.68 mg ml^−1^, which shows almost complete adsorption of protein is achieved. To obtain a comprehensive understanding of the adsorption behaviour of nano-Mg(OH)_2_, we further use the Langmuir and Freundlich models to assess the adsorptive ability.

The parameters of the Langmuir model (the saturated adsorption capacity *Q^0^*, adsorption affinity *b*) are used to characterize the adsorption ability for nano-Mg(OH)_2_. The saturated adsorption capacity of Cry protein was 100.2 mg g^−1^, and a higher capacity *Q^0^* of nano-Mg(OH)_2_ is beneficial for protein adsorption. Moreover, *b* is a constant related to the energy of adsorption that reflects the binding strength, and a higher value of *b* would also be a great help in adsorption. In most studies, several adsorbents have either large *Q^0^* with small *b*, or small *Q^0^* with large *b* [26]. But our results demonstrate that nano-Mg(OH)_2_ has large *Q^0^* and *b* values, which means that nano-Mg(OH)_2_ would be an excellent adsorbent in adsorption of Cry protein. Meanwhile, the value of the Freundlich constant *n* also illustrates that the adsorbate is favourably adsorbed.

The adsorption kinetics study indicated that the adsorption of protein was gradually increased, then approached the adsorption equilibrium after around 2 h. This means that the adsorption could be regulated in a short time in the practical application of nano-Mg(OH)_2_ as an adsorbent. Previous study also loaded the crystal protein anthelmintic Cry5B with partially oxidized mesoporous silicon (pSi), and the loading time was 16 h [17]. In our system, the adsorption of Cry11Aa by nano-Mg(OH)_2_ was a rapid process, and the adsorption capacity could achieve equilibrium in the first 2 h. This excellent adsorption ability might be attributed to the large adsorption affinity of nano-Mg(OH)_2_ and the relatively high BET areas. Additionally, the adsorption thermodynamic study indicated the exothermic nature of the adsorption; the Cry protein adsorption was more efficient at 30°C, but high temperatures are not suitable for adsorption. This result implied that temperature would be a critical factor in the practical application of nano-Mg(OH)_2_ as a protein carrier.

The adsorption experiments indicated that the Cry11Aa insecticidal protein could achieve good adsorption on nano-Mg(OH)_2_, and the adsorption process was driven not by electrostatic interaction but maybe by covalent linkage according to the zeta-potential result. Meanwhile, the FT-IR result showed decreased intensity of Mg-O (approx. 3700 cm^−1^), which might indicate that the surface Mg-O reacted and was changed; this finding would also support the zeta-potential result that there was covalent linkage between Cry protein and nano-Mg(OH)_2_.

Our further study on the insecticidal mechanism of the loaded material has indicated that nano-Mg(OH)_2_ could enhance the proteolysis of Cry protein in mosquito midgut and aggravate the damage to gut epithelial cells [23], and the enhanced toxicity could be attributed to the good adsorption of Cry protein on nano-Mg(OH)_2_. Therefore, a better understanding of the adsorption behaviour between Cry protein and nano-Mg(OH)_2_ could provide a basis for assessing nanoadsorbents, which would be a critical step in order to develop a new mosquitocide with high insecticidal activity.

## 5. Conclusion

In conclusion, this work explored the adsorption features between Cry11Aa and nano-Mg(OH)_2_. The results indicated that the adsorption process is well fitted to Langmuir and Freundlich isotherm models and followed pseudo-first or -second order rate models; the adsorption was spontaneous and an exothermic process, and high temperatures are not suitable for adsorption. Moreover, the Cry11Aa protein was loaded onto the nano-Mg(OH)_2_ perhaps through intermolecular forces instead of electrostatic adsorption, and the secondary structure of the protein was changed during the adsorption process. Our work provided details of the adsorption process, which would offer the basic information for use of nano-Mg(OH)_2_ as a nano-carrier in a practical application.

## Data accessibility

All original data are deposited at the Dryad Digital Repository: http://dx.doi.org/10.5061/dryad.08v2c [33].
